# White blood cell count affects fetal fraction and test failure rates in noninvasive prenatal screening

**DOI:** 10.3389/fmed.2023.1088745

**Published:** 2023-02-02

**Authors:** Longwei Qiao, XiaoJu Cao, Haoyu Tang, Zheng Yu, Jingye Shi, Ying Xue, Ting Wang, Yuting Liang, Chao Huang, Jiucun Wang

**Affiliations:** ^1^State Key Laboratory of Genetic Engineering, School of Life Sciences, and Human Phenome Institute, Fudan University, Shanghai, China; ^2^Collaborative Innovation Center for Genetics and Development, Fudan University, Shanghai, China; ^3^Suzhou Municipal Hospital, Center for Reproduction and Genetics, School of Gusu, The Affiliated Suzhou Hospital of Nanjing Medical University, Suzhou, China; ^4^Center for Clinical Laboratory, The First Affiliated Hospital of Soochow University, Suzhou, Jiangsu Province, China

**Keywords:** noninvasive prenatal screening, cell-free DNA, fetal fraction, WBC count, multivariate regression models

## Abstract

**Objective:**

To investigate the effects of white blood cell (WBC) count on fetal fraction (FF), which is an essential quality control for obtaining reliable results, and on the rate of screen failures in noninvasive prenatal screening (NIPS).

**Methods:**

Noninvasive prenatal screening, serum lipid and liver enzyme level measurements, and WBC count were performed for 4,281 pregnancies with male fetuses. After adjusting for confounders, including the maternal characteristics and alanine aminotransferase (ALT) levels, the effect of WBC count on FF and test failure rate was measured by linear and logistic regression analyses.

**Results:**

Fetal fraction was negatively associated with BMI, ALT, IVF conceptions, and WBC count and positively correlated with gestational age in the multivariate linear regression model. Moreover, WBC count was the most important factor affecting FF after BMI according to the standardization coefficient analysis. In the 4,281 pregnancy samples with male fetuses, FF decreased with WBC count from 11.45% at ≤8 to 9.02% at >12, and FF markedly decreased to 7.40% in pregnancies with a higher WBC count (>12) and higher BMI (≥25 kg/m^2^). Meanwhile, the test failure rates were significantly higher in the WBC count > 12 group (4.29%) than in the WBC count ≤ 8 group (0.89%). Notably, when the BMI of pregnancies with a WBC count of >12 was >25, the rate reached 7.53%. Subsequently, multivariate logistic regression analysis further confirmed that an increased BMI and WBC count were independently and significantly associated with the test failure rates.

**Conclusion:**

An increased WBC count was associated with lower FF and higher test failure rates, suggesting that these important factors should be carefully considered during genetic counseling in pregnant women who decide to undergo blood collection or resampling.

## Introduction

Cell-free DNA (cfDNA) testing can be used not only to screen for trisomies 21, 18, and 13 but also to expand the detection of sex chromosome anomalies, rare autosomal anomalies, and sub-chromosomal copy number variants (1, 2). Fetal fraction (FF) is the percentage of the total plasma cfDNA and is also a key quality control parameter to ensure high sensitivity and specificity of the cfDNA test. Theoretically, the higher the FF, the more reliable it is to distinguish between aneuploid and euploid pregnancies. A lower FF or even below the lower limit of laboratory detection can lead to screen failures and increase the risk of false-negative results (3). Thus, it is particularly important to obtain adequate fetal cfDNA.

Previous studies have shown that fetal-derived cfDNA tends to be shorter than maternal-derived cfDNA, suggesting that shorter cfDNA fragments have the potential to enrich FF (2, 4–6). Indeed, this new enrichment technique can significantly improve FF and reduce test failures and false negatives (2, 4–6). However, this new technology requires additional enrichment costs and has not been used in clinical practice. Moreover, other clinical studies have examined the effects of biological factors, including gestational age, BMI, triglyceride (TG), and alanine aminotransferase (ALT), on FF, with the aim of controlling these factors during pregnancy to decrease the maternal contribution and/or increase the placental contribution and to obtain higher fetal-derived cfDNA (7–10). These strategies are interesting and cost-effective. Notably, our previously study indicated that the FF and the rate of test failures in NIPT are affected by TG levels and delay in the time between NIPT blood collections can significantly reduce the impact of TG (10). We also found that ALT is another important factor that affects screening failure rates, especially when IVF pregnant women have higher ALT (>40 U/L), the test failure rate reaches 9.52% (9). Adjusting these new factors is conducive to the quality control before NIPT testing and reduces testing failures. However, the test failure rate caused by a low FF is still high (approximately 1%) (11), indicating that there may be other key factors that have not been identified. Interestingly, the hematopoietic system (mainly white blood cells) has been reported to be the predominant source of maternal-derived DNA (12, 13). Although white blood cells (WBCs) are an important component of plasma cfDNA, so far, there are no clinical data to study how they affect FF.

In the present study, which analyzed 4,281 pregnancies wherein cfDNA sequencing, pregnancy patterns (IVF or spontaneous conceptions), lipid metabolism, white blood cell count, and liver enzyme level measurement were performed, we aimed (1) to investigate the potential effects of white blood cells on FF and (2) to evaluate whether adjusting these factors can help reduce the rate of screen failures.

## Materials and methods

### Study population

A total of 4,281 pregnancy samples with male fetuses were collected at the prenatal diagnostic centers of Suzhou Municipal Hospital between January 2016 and December 2021 after approval by the Reproductive Medicine Ethics Committee of Suzhou Municipal Hospital (ID: K2021032H01) for a retrospective cohort study. This ethical approval is a nested case–control study of maternal characteristics and relevant laboratory parameters, including lipid metabolism, ALT, and WBC count, that affect FF and the rate of screen failures of noninvasive prenatal screening (NIPS), as described in our previous study (9, 10). In this study, we mainly identified the new influencing factor, WBC count, on fetal fraction and the rate of screen failures in NIPT. When the two previous studies were published, the number of pregnant women with WBC count detection was insufficient. In addition, in recent clinical practice, we found that WBC count also rose when some patients failed to test, which prompted this study. The study inclusion criteria were as follows: (1) all pregnancies underwent genetic counseling; (2) maternal age, gestational age, and BMI were recorded; (3) all samples underwent cfDNA testing; and (4) WBC count and ALT and TG levels were available.

### Sample testing and data collection

Maternal serum samples were collected after 6–8 h restrained eating and analyzed for ALT, WBC count, and TG using Beckman Coulter AU5800 (Beckman Coulter, Brea, CA, United States), Mindray BC-6800 Plus automated hematology analyzer, and COBAS INTEGRA® 400 plus analyzer (Roche Diagnostics, Indianapolis, IN, United States), respectively.

All blood samples were collected in EDTA tubes following pretest counseling. cfDNA was extracted from 200 μl of plasma using a QIAamp DSP DNA Blood Mini Kit (Qiagen) (14). Library construction and sequencing were performed using the BGISEQ-500 platform (MGI, China). FF was evaluated by calculating the number of Y chromosome reads. A low FF (usually <4%) can result in screen failure. All NIPS-positive pregnancies had fetal karyotype or clinical follow-up results.

### Statistical analysis

The primary outcomes were FF and test failures of NIPS according to WBC count. The secondary outcomes were FF and test failures in different WBC count subgroups according to BMI or IVF conception.

Descriptive data are presented as the median and interquartile range for non-normally distributed continuous variables. Fetal fraction was the square root (√) transformed to obtain a Gaussian distribution. The associations between FF and BMI, ALT, IVF conceptions, WBC count, and gestational age were assessed using univariate and multivariate linear regression analyses. The variance inflation factor was used to evaluate multicollinearity, and values > 10 suggest severe multicollinearity and need to be corrected. WBC counts were categorized into four groups: ≤8, 8.01–10, 10.01–12, and >12 (×10^9^/L). BMI was categorized as <25 and ≥25 kg/m^2^. Three linear models were used to further test the independent association between the WBC count categories and FF. Model 1 was a univariate linear regression of the relationship between the WBC count and FF. Model 2 was adjusted for BMI, IVF conception, and gestational age. Model 3 added ALT to the model on the basis of model 2.

The association between test failure and BMI, ALT, IVF conceptions, and WBC count was assessed using univariate and multivariate logistic regression models. Three models were used to assess the impact of each WBC count category on test failure. Model 1 was a univariate logistic regression analysis of the relationship between the WBC count and test failure rate. Model 2 was adjusted for BMI. Model 3 ALT was based on model 2. SPSS version 26.0 (IBM Corp., Armonk, NY, United States) was used for statistical analysis. All *p* values were two-sided, and statistical significance was set at *p* < 0.05.

## Results

### Sample characteristics

The general characteristics of the pregnant women are summarized in Table 1. Among the 4,281 pregnancies with male fetuses enrolled in this study, the median maternal age, gestational age, FF, BMI, ALT level, TG level, and WBC count were 31 years (range, 28–34 years), 16.6 weeks (range, 15.9–17.4 weeks), 10.47% (range, 8.00–13.42%), 22.20 kg/m^2^ (range, 20.50–24.40 kg/m^2^), 15 U/L (range, 11–22 U/L), 1.42 mmol/L (range, 1.10–1.85 mmol/L), and 9.25 × 10^9^/L (range, 7.93–10.66 × 10^9^/L), respectively. In addition, 880 (20.6%) pregnant women were overweight or obese (BMI, ≥25 kg/m^2^), and 486 (11.4%) underwent IVF.

**Table 1:** Sample characteristics of the study population (*n* = 4,281)

Characteristic	Value (median and interquartile range)
Age (year)	31 (28–34)
Gestational age (week)	16.6 (15.9–17.4)
FF (%)	10.47 (8.00–13.42)
BMI (kg/m^2^)	22.20 (20.50–24.40)
ALT (U/L)	15 (11–22)
TG (mmol/L)	1.42 (1.10–1.85)
WBC (10^9^/L)	9.25 (7.93–10.66)

FF, fetal fraction; ALT, alanine aminotransferase; TG, triglyceride; and WBC: white blood cell.

### Multiple factors affecting FF

**Figure 1:**
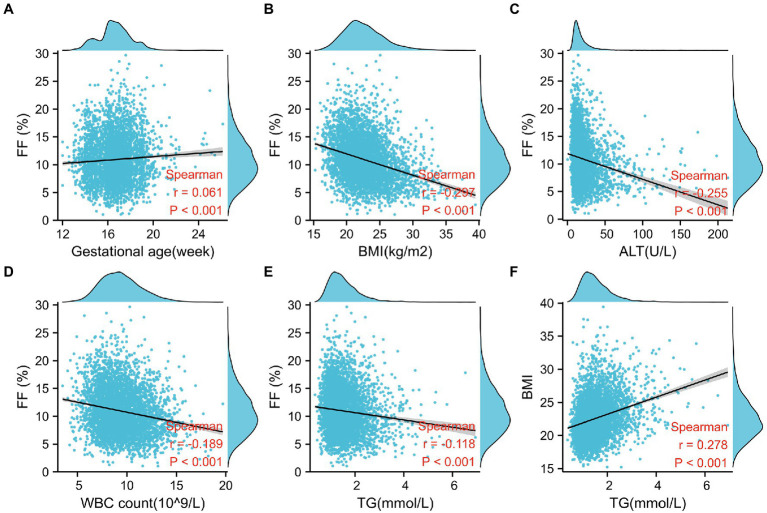
Factors associated with fetal fraction (FF) in noninvasive prenatal testing. **(A)** Gestational age is positively correlated with FF. **(B–E)** FF decreases with a higher BMI, alanine aminotransferase level (ALT), white blood cell (WBC) count, and TG level. **(F)** TG is positively correlated with BMI.

Using Spearman’s correlation, FF was negatively associated with BMI (Spearman *r* = −0.297, *p* < 0.001; Figure 1B), ALT (Spearman *r* = −0.255, *p* < 0.001; Figure 1C), WBC count (Spearman *r* = −0.189, *p* < 0.001; Figure 1D), and TG (Spearman *r* = −0.118, *p* < 0.001; Figure 1E), whereas it was weakly and positively correlated with gestational age (Spearman *r* = 0.061, *p* < 0.001; Figure 1A). Moreover, scatterplots suggest that the aforementioned factors have a linear relationship with FF. Univariate or multivariate linear regression analyses were performed to determine the factors that influence FF. Univariate linear regression analysis demonstrated that √FF was negatively correlated with maternal age (regression coefficient = −0.012, *p* < 0.001), BMI (regression coefficient = −0.061, *p* < 0.001), TG (regression coefficient = −0.106, *p* < 0.001), ALT (regression coefficient = −0.006, *p* < 0.001), IVF conceptions (regression coefficient = −0.237, *p* < 0.001), and WBC count (regression coefficient = −0.057, *p* < 0.001) and positively correlated with gestational age (regression coefficient = 0.023, *p* < 0.001). As maternal BMI was related to TG (*r* = 0.278, *p* < 0.001), it was not included in the multiple regression analysis (Figure 1F; Table 2).

**Table 2:** Regression analysis for predicting √FF in 4,281 pregnancies with male fetuses.

Independent variable	Univariable		Multivariable		
	Regression coefficient (95%CI)	*p*	Regression coefficient (95%CI)	Standardized coefficients	*p*
Age	−0.012 (−0.016 to −0.007)	<0.001	−	−	−
BMI (kg/m^2^)	−0.061 (−0.066 to −0.055)	<0.001	−0.053 (−0.058 to −0.047)	−0.268	<0.001
Gestational age (week)	0.023 (0.011–0.035)	<0.001	0.033 (0.022–0.044)	0.084	<0.001
IVF conceptions	−0.237 (−0.295 to −0.180)	<0.001	−0.171 (−0.225 to −0.118)	−0.089	<0.001
TG	−0.106 (−0.133 to −0.079)	<0.001	−	−	−
ALT(U/L)	−0.006 (−0.007 to −0.005)	<0.001	−0.004 (−0.005 to −0.004)	−0.144	<0.001
WBC (10^9^/L)	−0.057 (−0.065 to −0.048)	<0.001	−0.043 (−0.051 to −0.035)	−0.146	<0.001

ALT, alanine aminotransferase; TG, triglyceride; and WBC, white blood cell.

**Figure 2:**
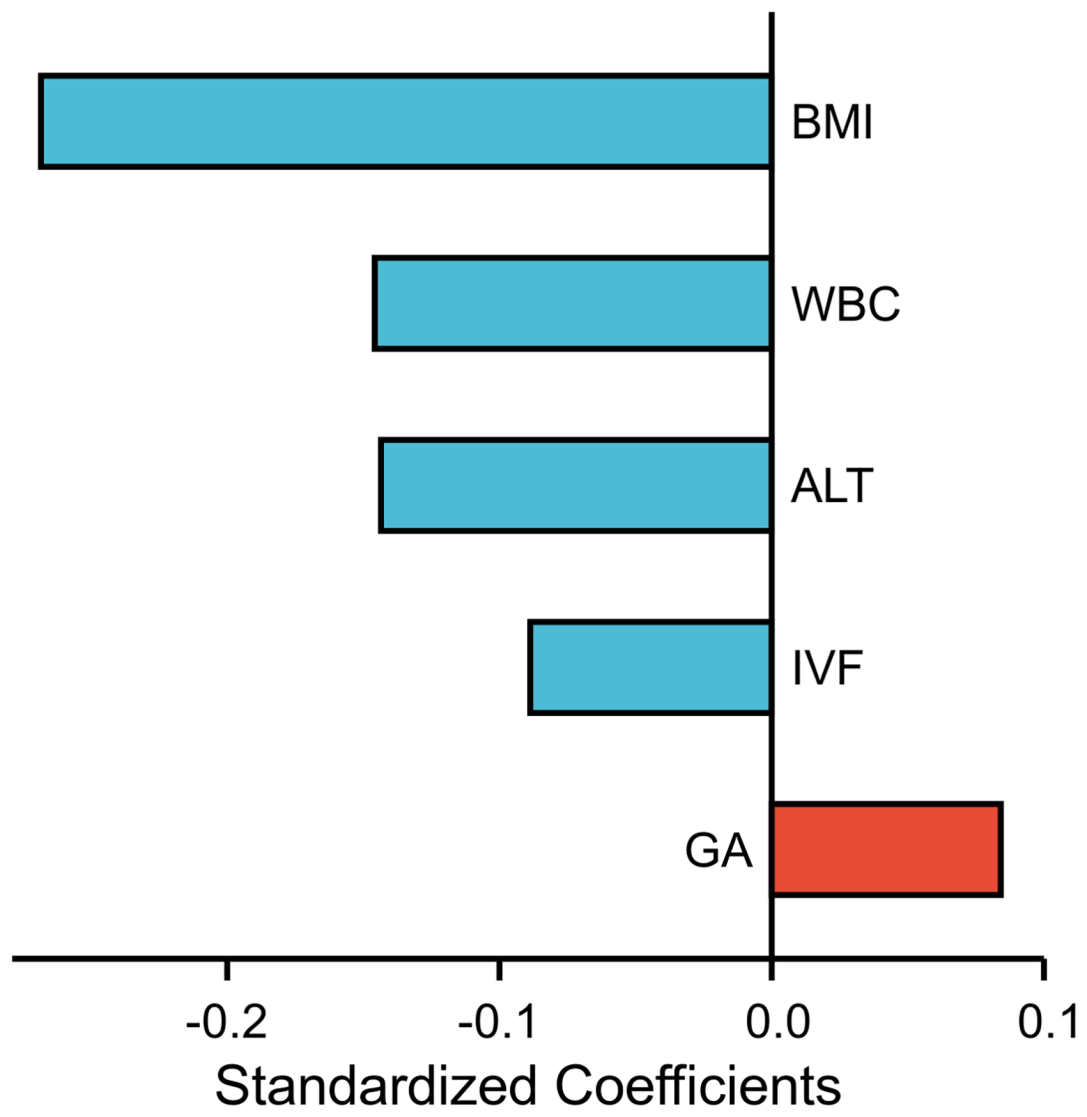
Each factor’s contribution in the multivariate linear regression analysis is measured by standardized coefficients.

Subsequently, in the multivariate linear regression model, the associations between FF and BMI (regression coefficient = −0.053, *p* < 0.001), ALT (regression coefficient = −0.004, *p* < 0.001), IVF conceptions (regression coefficient = −0.171, *p* < 0.001), WBC count (regression coefficient = −0.043, *p* < 0.001), and gestational age (regression coefficient = 0.033, *p* < 0.001) were maintained. Moreover, a standardized coefficient represented the contribution of the factor in the multivariate linear regression analysis. As shown in Figure 2, the WBC count was the most important factor affecting FF after BMI.

### FF decreases with increasing WBC count

**Figure 3:**
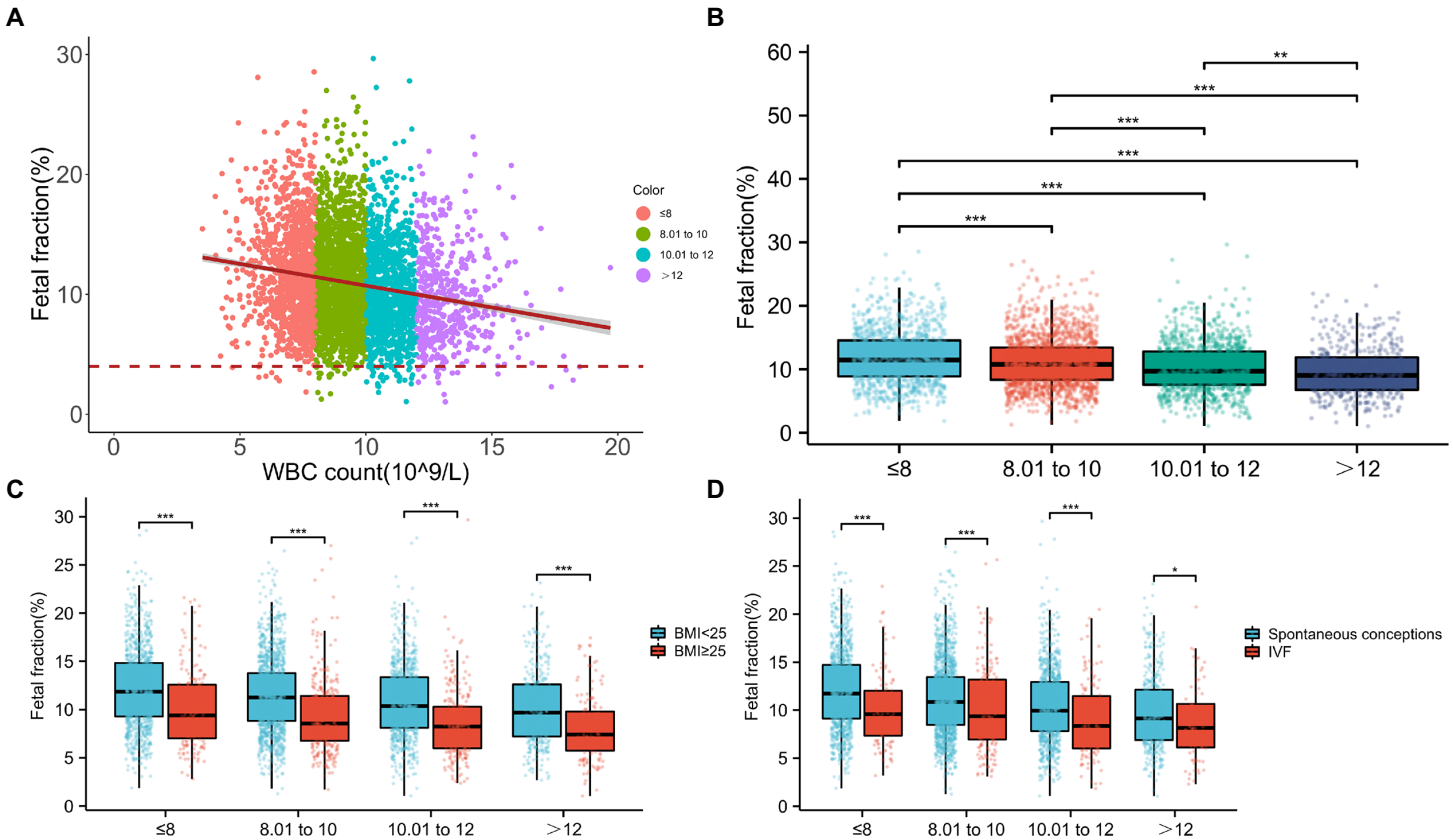
Relationship between fetal fraction (FF) and white blood cell (WBC) count. **(A)** Scatterplots of FF according to WBC count categories. **(B)** The median FFs across WBC count categories are 11.45, 10.77, 9.67, and 9.02, respectively. **(C)** In the same WBC count categories, FF decreases with a higher BMI. Similarly, in the same BMI categories, FF decreases with WBC count. **(D)** In the same WBC count categories, FFs are significantly higher in spontaneous conceptions than in IVF conceptions. Similarly, in the same mode of pregnancy, FF decreases with the WBC count.

To further study, the relationship between WBC count and FF and facilitate clinical application, pregnancies were categorized into four groups according to WBC count: ≤8, 8.01–10, 10.01–12, and >12 (×10^9^/L). The numbers of participants with WBC counts in these four classifications were 1,124, 1,640, 1,027, and 490, respectively. Scatterplots showed that FF decreased with a higher WBC count (Figure 3A). Indeed, the median FFs across WBC count classifications were 11.45% (range, 8.89–14.54%), 10.77% (range, 8.33–13.42%), 9.67% (range, 7.57–12.81%), and 9.02% (range, 6.74–11.88%) (Figure 3B) and tended to decrease gradually (Figure 3B).

Moreover, considering that the number of samples in each subgroup was not too small, we classified different WBC count subgroups according to BMI or IVF conceptions. As shown in Figures 3C,D, in the same WBC count categories, FF decreased with a higher BMI or IVF conceptions. Similarly, in the same BMI categories or IVF conceptions, FF decreased with the WBC count. In particular, compared with lower WBC count (≤8) with spontaneous conceptions, the median FF was 8.14% and decreased in the higher WBC count pregnancies (>12) with IVF conceptions. In addition, the median FF was 7.40% and markedly decreased in pregnancies with a higher WBC count (>12) and higher BMI (≥25 kg/m^2^) compared with that in normal pregnancies (WBC count ≤ 8, BMI < 25 kg/m^2^), suggesting that pregnancies with a higher WBC count (>12) and higher BMI (≥25 kg/m^2^) were more inclined to have lower FF and higher test failures.

**Figure 4:**
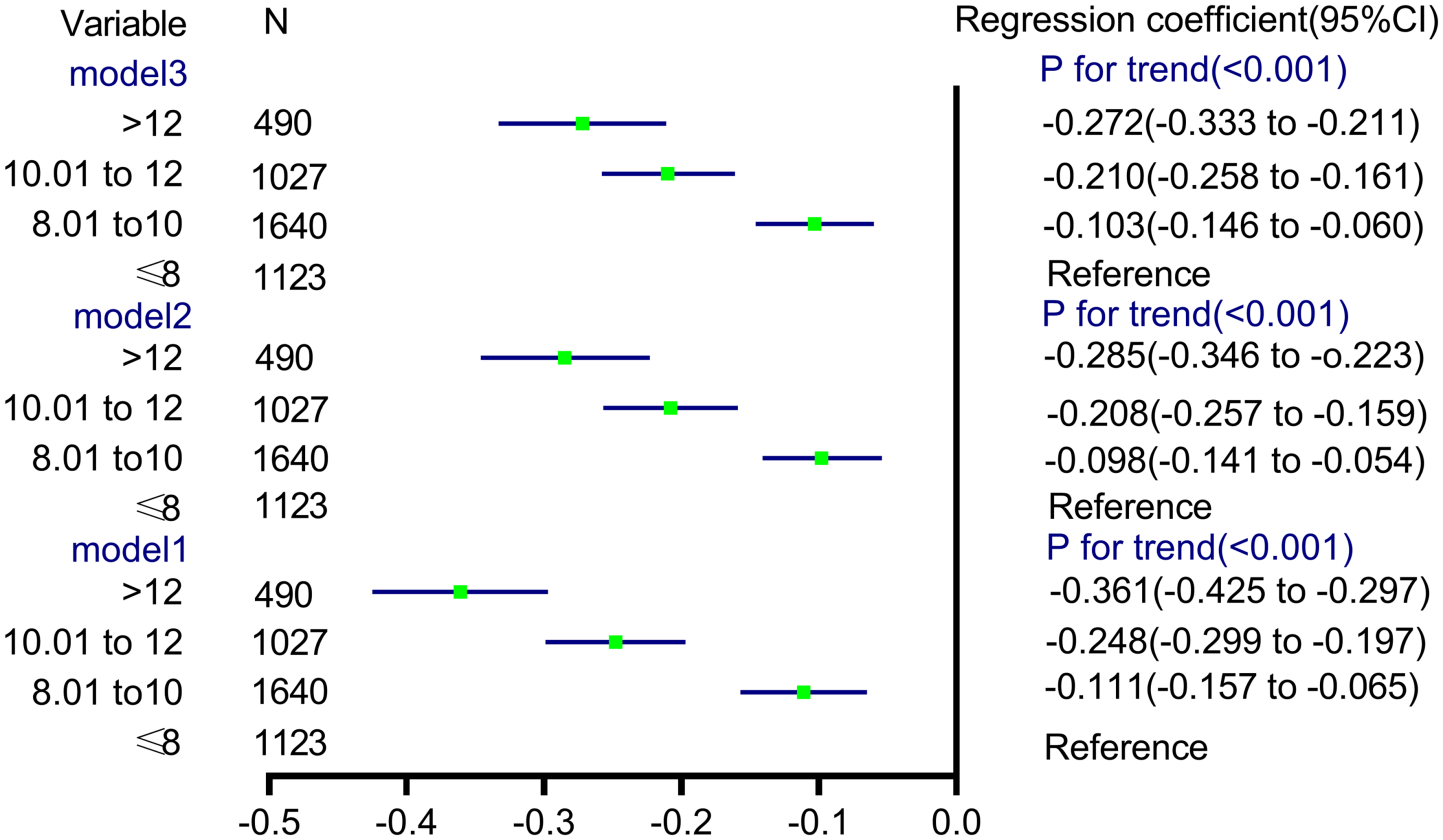
Differences in √fetal fraction according to white blood cell count categories.

We also used a general linear model to further test the independent association between WBC count categories and FF (Figure 4). In unadjusted model 1, there was a statistically significant inverse correlation between the categories of WBC count and mean FF (model 1, P*_trend_* < 0.001). Further adjustment for gestational age, BMI, ALT, and IVF conceptions showed similar tendencies (model 3, *p_trend_* < 0.001).

### WBC count affects the test failure rates of NIPS

Among the full pregnancies, 78 (1.82%) had a test failure rate of NIPS. Univariate regression analysis showed that a higher test failure rate was associated with an increased BMI [odds ratio (OR), 1.172; 95% CI, 1.106–1.243], ALT (OR, 1.006; 95% CI, 1.001–1.012), WBC count (OR, 1.272; 95% CI, 1.157–1.399), and IVF conceptions (OR, 3.378; 95% CI, 2.057–5.548) but not with gestational age (OR, 0.979; 95% CI, 0.845–1.133; *p* < 0.001). Moreover, these associations remained statistically significant in multivariate logistic regression analysis (Table 3).

**Table 3:** Logistic regression analysis factors that contribute to the test failure rate

	Univariable		Multivariable	
Independent variable	Odds ratio (95% CI)	*p*	Odds ratio (95% CI)	*p*
BMI (kg/m^2^)	1.172 (1.106–1.243)	<0.001	1.141 (1.074–1.213)	<0.001
Gestational age (week)	0.979 (0.845–1.133)	0.771	−	−
IVF conceptions	3.378 (2.057–5.548)	<0.001	2.844 (1.715–4.714)	<0.001
ALT (U/L)	1.006 (1.001–1.012)	0.025	1.006 (1.000–1.012)	0.054
WBC (10^9^/L)	1.272 (1.157–1.399)	<0.001	1.208 (1.096–1.330)	<0.001

**Figure 5:**
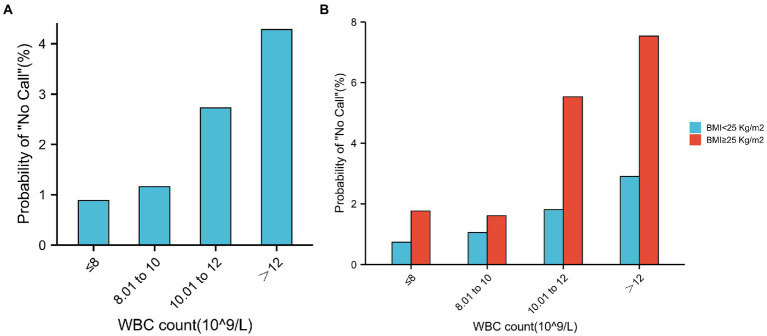
Test failure rate according to the different groups of WBC count. **(A)** Relationship between test failure rate and different groups of WBC count. **(B)** In the same WBC count categories, test failure rate increases with a higher BMI. Similarly, in the same BMI categories, test failure rate increases with WBC count.

We then determined the test failure rates in each subgroup of the WBC count. Although still low, the test failure rates were significantly higher in the WBC count > 12 group (4.29%) than in the WBC count ≤ 8 group (0.89%; Figure 5A). Meanwhile, when the BMI of pregnancies with a higher WBC count (>12) was greater than 25, the test failure rate significantly increased to 7.53% (Figure 5B).

**Figure 6:**
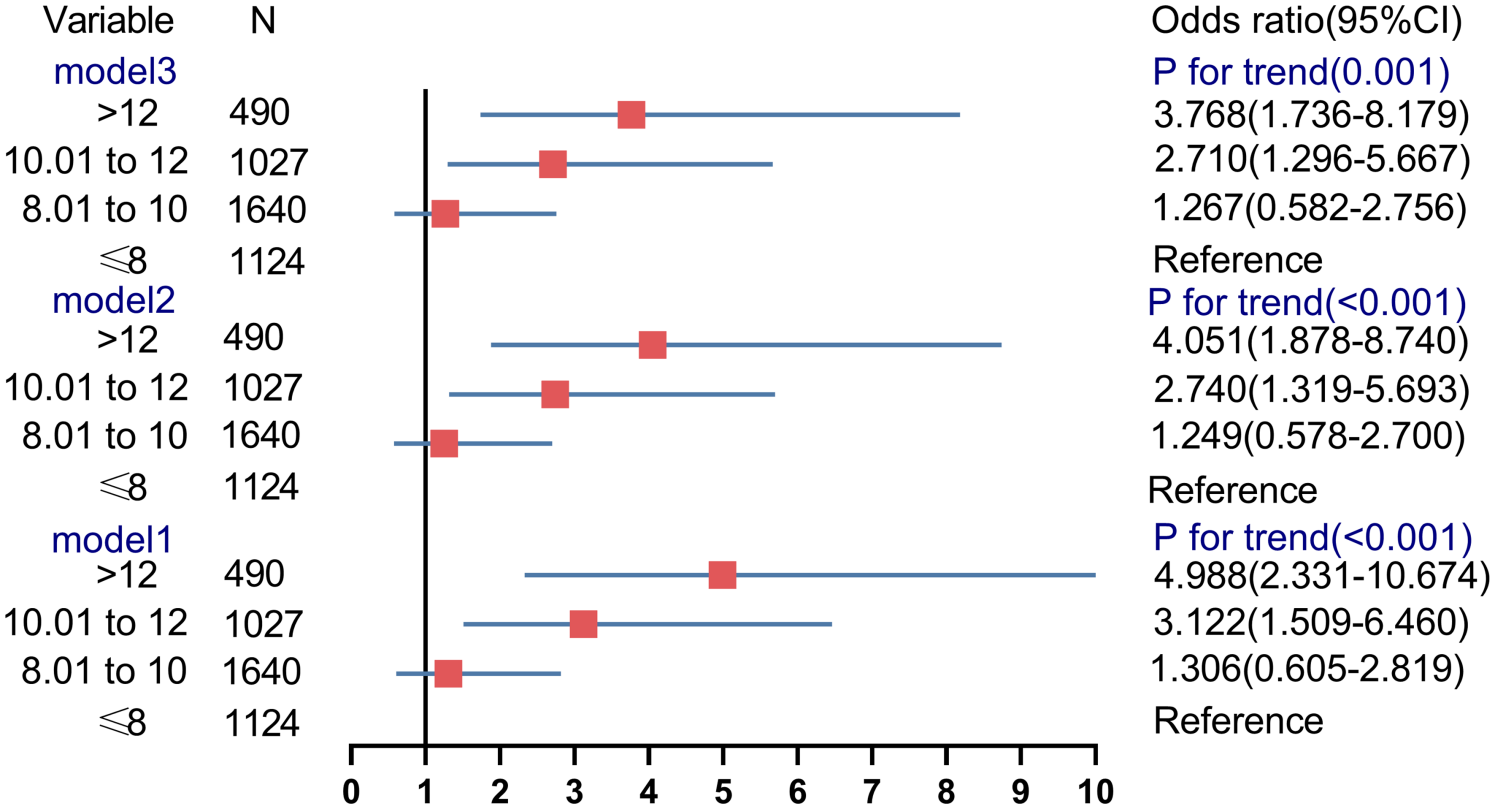
Association between the different groups of white blood cell count and risk of noninvasive prenatal screening failures.

Logistic regression analysis was performed to estimate the OR of the test failure rates based on the WBC count (Figure 6). In unadjusted model 1, pregnancies with WBC counts of 10.01–12 and >12 had an increased risk of test failure rates (OR, 3.1224; 95% CI, 1.509–6.460, and OR, 4.988; 95% CI, 2.331–10.674, respectively). Moreover, compared with women with a WBC count of ≤8, the confounder-adjusted OR (model 3) of the test failures still increased with a higher WBC count (>12; OR, 3.768; 95% CI, 1.736–8.179; *p_trend_* < 0.001).

## Discussion

The findings of lower FF and increased screen failure rates in pregnancies with a higher WBC count (>12) and higher BMI have implications for optimal pretest counseling and clinical management provided to those women. To the best of our knowledge, this is the most extensive study to determine the effects of BMI, pregnancy patterns, lipid metabolism, ALT, and WBC count on the FF and test failure rate in the cfDNA test. Our results suggest that FF was negatively associated with BMI, ALT, IVF conceptions, and WBC count and positively correlated with gestational age. Notably, the WBC count was the most important factor affecting FF after BMI. Moreover, the median FF was lower (7.40 vs. 11.85%), and the test failure rate was markedly higher (7.53 vs. 0.73%) in pregnancies with a higher WBC count (>12) and higher BMI (≥25 kg/m^2^) than in normal pregnancies (WBC count, ≤8; BMI, <25 kg/m^2^), suggesting that pregnancies with a higher WBC count (>12) with higher BMI (≥25 kg/m^2^) were the focus of pretest counseling. Furthermore, these associations remained statistically significant in the multivariate regression analysis.

A possible explanation could be that the maternal cfDNA contribution increased, causing a relative reduction in FF. As the most well-recognized factor of FF, BMI negatively associated with FF is thought to be due to adipocyte inflammation and necrosis, resulting in an increased release of maternal origin cfDNA into the circulation and overcoming the dilutional effect that occurs in obese pregnant women (15). The cfDNA of a pregnant woman is a mixture of DNA from blood cells; placenta; fetus; viruses; solid organs, including the liver, solid tumors, and many other sources (1). Meanwhile, cfDNA derived from the white-cell lineage contributes more than 70–90% (12, 13, 16). It is not difficult to speculate that an increase in the WBC count will reduce FF, and this important factor is easily ignored by clinicians. When the superposition of the aforementioned two factors lowered the FF, this was confirmed in our data. It has also been shown that liver-derived cfDNA released from dying hepatocytes is positively correlated with the ALT level, which reflects liver damage (17). In particular, pregnancies with ALT levels of >40 U/L have a higher test failure rate (9). IVF conceptions are another important factor affecting lower FF. This may be the result of shallow placentation and increased inflammation in IVF conceptions (18, 19).

When a pregnant woman receives a test failure result, professional genetic counseling is strongly recommended. Pregnancy should be advised that test failure may be influenced by biological factors affecting FF or may imply a higher risk of fetal aneuploidy (20). Blood redrawn for the cfDNA test while reducing WBC count, such as reduce the physiological WBC count increase caused by excessive exercise and treat infection, and liver damage may benefit these women. However, this strategy still has a failure rate of 40–50%, and with the increase in gestational weeks, it will affect the decision of invasive prenatal diagnosis and increase maternal anxiety (3). Based on the FF measured at the initial sample, women’s weight, and the interval between blood collections, a personalized estimate of the likelihood of the test success rate of resampling could be considered (21). If the test fails following resampling, prenatal diagnosis and regular ultrasonography are recommended. Moreover, size selection NIPS as an alternative prenatal screening may be an option (4–6).

Together, we have, for the first time, identified WBC count as an important biological factor influencing FF and test failure rate after adjusting for other confounders in NIPS. FF was lower, and the test failure rate was higher in pregnancies with a higher WBC count (>12) and higher BMI (≥25 kg/m^2^) than in normal pregnancies (WBC count, ≤8; BMI, <25 kg/m^2^), suggesting that these limitations should be carefully considered during genetic counseling in pregnant women who decide to undergo blood collection or resampling.

## Data availability statement

The original contributions presented in this study are included in the article, further inquiries can be directed to the corresponding author. Data are available upon reasonable request.

## Ethics statement

This study was approval by the Reproductive Medicine Ethics Committee of Suzhou Municipal Hospital (ID: K2021032H01). Written informed consent was not required to participate in this study in accordance with the national legislation and the institutional requirements.

## Author contributions

LQ and XC: conception, design, collection, and assembly of data. YX and HT: administrative support. ZY, TW, and JS: provision of study materials or patients. CH, YL, and JW: data analysis and interpretation. LQ, XC, HT, ZY, JS, YX, TW, YL, CH, and JW: manuscript writing and final approval of manuscript. All authors contributed to the article and approved the submitted version.

## Funding

This study was supported by the National Natural Science Foundation of China (Grant Nos. 82001576 and 81901632), Suzhou Science and Technology Support Program (SYS2019095, SYS2019098, BE2019683, and SS2019066), Jiangsu Maternal and Children Health Care Key Discipline (FXK202142), the Primary Research & Development Plan of Jiangsu Province (BE2022736), and Shanghai Municipal Science and Technology Major Project (2017SHZDZX01).

## Conflict of interest

The authors declare that the research was conducted in the absence of any commercial or financial relationships that could be construed as a potential conflict of interest.

## Publisher’s note

All claims expressed in this article are solely those of the authors and do not necessarily represent those of their affiliated organizations, or those of the publisher, the editors and the reviewers. Any product that may be evaluated in this article, or claim that may be made by its manufacturer, is not guaranteed or endorsed by the publisher.
